# Increasing Brain Permeability of PHA-767491, a Cell Division Cycle 7 Kinase Inhibitor, with Biodegradable Polymeric Nanoparticles

**DOI:** 10.3390/pharmaceutics13020180

**Published:** 2021-01-28

**Authors:** Elisa Rojas-Prats, Carlota Tosat-Bitrián, Loreto Martínez-González, Vanesa Nozal, Daniel I. Pérez, Ana Martínez

**Affiliations:** 1 Centro de Investigaciones Biológicas Margarita Salas-CSIC, Ramiro de Maeztu 9, 28040 Madrid, Spain; ely.rp.91@gmail.com (E.R.-P.); carlota.tosat@cib.csic.es (C.T.-B.); loretomg@cib.csic.es (L.M.-G.); vanesanozal@cib.csic.es (V.N.); 2 Centro de Investigación Biomédica en Red de Enfermedades Neurodegenerativas (CIBERNED), Instituto de Salud Carlos III, 28031 Madrid, Spain

**Keywords:** ALS, TDP-43, CDC7 inhibitor, PHA-76749, nanoparticle, PLGA, nanoprecipitation, BBB

## Abstract

A potent cell division cycle 7 (CDC7) kinase inhibitor, known as PHA-767491, has been described to reduce the transactive response DNA binding protein of 43 KDa (TDP-43) phosphorylation in vitro and in vivo, which is one of the main proteins found to aggregate and accumulate in the cytoplasm of motoneurons in amyotrophic lateral sclerosis (ALS) and frontotemporal dementia (FTD) patients. However, the main drawback of this compound is its low permeability to the central nervous system (CNS), limiting its use for the treatment of neurological conditions. In this context, the use of drug delivery systems like nanocarriers has become an interesting approach to improve drug release to the CNS. In this study, we prepared and characterized biodegradable nanoparticles in order to encapsulate PHA-767491 and improve its permeability to the CNS. Our results demonstrate that poly (lactic-co-glycolic acid) (PLGA) nanoparticles with an average radius between 145 and 155 nm could be used to entrap PHA-767491 and enhance the permeability of this compound through the blood–brain barrier (BBB), becoming a promising candidate for the treatment of TDP-43 proteinopathies such as ALS.

## 1. Introduction

**Figure 1:**
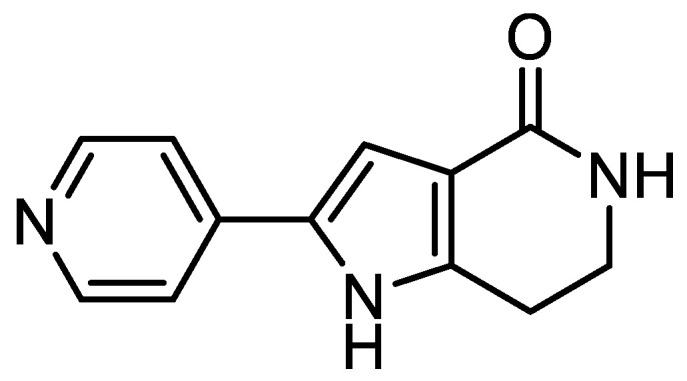
Chemical structure of cell division cycle 7 (CDC7) inhibitor PHA-767491 (PHA).

The small molecule known as PHA-767491 (PHA) was one of the first representative cell division cycle 7 (CDC7) kinase inhibitors described [1]. This inhibitor of low molecular weight has a half-maximal inhibitory concentration (IC_50_) of 10 nM on CDC7 and has been characterized as an ATP-competitive kinase inhibitor (Figure 1). It has demonstrated a great antitumor activity in different cancer cell lines and in rodents, showing its potential as an anticancer drug [2]. For this reason, PHA has reached clinical trials for the treatment of solid tumors and advanced/metastatic solid tumors [3].

In the last years, PHA has gained attention because it is the first CDC7 inhibitor described to prevent neurodegeneration driven by pathological phosphorylation of the key transactive response DNA-binding protein of 43 kDa (TDP-43) both in vitro and in vivo [4]. TDP-43 is a nuclear protein encoded by *TARDBP* gene that regulates several RNA processes as transcription, mRNA transport and microRNA biosynthesis [5]. In normal conditions, it is located in the nucleus of the cells but in pathological conditions, such as in amyotrophic lateral sclerosis (ALS) or frontotemporal dementia (FTD), TDP-43 is translocated to the cytoplasm where suffers from multiple post-translational modifications including hyperphosphorylation, ubiquitination and truncation that lead to its accumulation [6]. In 2013, Liachko et al. demonstrated that CDC7 was able to directly phosphorylate TDP-43 and tested PHA in different cellular and animal models in order to see the effects of this CDC7 inhibitor in TDP-43 phosphorylation [4]. They found that the treatment of motor-neuron-enriched NSC-34 cells with 10 μM of PHA prevented TDP-43 phosphorylation. PHA was then assayed in a *C. elegans* transgenic model carrying a human TDP-43 mutation, where it was not only able to reduce TDP-43 phosphorylation but also prevent neuronal death in treated animals. Thus, PHA seems to be a good candidate for the treatment of TDP-43 proteinopathies. However, the main drawback of this compound is its low permeability to the central nervous system (CNS) which limits its use for the treatment of neurological conditions such as ALS [2].

One of the main problems in drug development for neurodegenerative diseases is that about 50% of the small molecules designed and synthesized in the laboratory are poorly permeable through the blood–brain barrier (BBB) [7], and such is the case for PHA. The BBB is a physical barrier between the brain and the blood vessels. It is made of highly specialized endothelial cells joined by tight cell-to-cell junctions and a basement membrane [8]. These endothelial cells are also surrounded by different cellular elements including pericytes and astrocytes forming an additional membrane that protect the CNS from external agents. Its main function is to regulate the transport of endogenous and exogenous substances between the blood and the brain like glucose, amino acids and proteins [9]. The majority of molecules cross the BBB by passive diffusion while others need specific membrane transporters. In this context, several structural modifications have been made in order to improve PHA cell permeability unsuccessfully [10].

In the last two decades, the development of drug delivery systems has become an attractive tool for the efficient transport of drugs and their controlled release to the target tissue [11]. In this way, some of the main problems associated with conventional drug administration methods, like unspecific distribution, rapid metabolism or low bioavailability, can be avoided. Among different drug delivery systems, nanoparticles (NPs) have been widely studied for their unique chemical, physical and biological properties as well as their clinical applications [12]. Regarding their composition, NPs can be classified as organic or inorganic nanoparticles [13], and many of them, especially liposomes and polymeric NPs, have been approved for the treatment of different pathologies, such as Doxil^®^ (Janssen, 1995) or Plegridy^®^ (Biogen, 2014) for the therapy of ovarian cancer and multiple sclerosis, respectively [14,15].

There is a special interest in polymeric nanoparticles due to their biocompatibility with cells and tissues and stability in blood [16]. Among the different polymers that can be used for nanoparticle formulation, poly (d,l-lactide-co-glycolide) (PLGA) is one of the most frequently used synthetic polymers and one of the few approved by the Food and Drug Administration (FDA) for clinical uses such as sutures or tissue engineering [17]. This is mainly because it undergoes hydrolysis to produce lactic and glycolic acids which are naturally metabolized in the body. Moreover, PLGA nanoparticles can be used to encapsulate and transport hydrophobic and non-BBB permeable drugs, improving their controlled release to the CNS [18]. For these reasons, PLGA nanoparticles have been widely studied for the delivery of therapeutics for several pathologies including cancer, anti-inflammatory and cardiovascular disorders and neurological diseases [19]. Therefore, PLGA nanoparticles are considered as an optimal method to improve PHA BBB permeation.

In this work, we prepared and characterized biodegradable nanoparticles based on a PLGA copolymer matrix in order to encapsulate PHA as a novel strategy to improve its permeability and delivery to the CNS for the treatment of neurodegenerative disorders, mainly TDP-43 proteinopathies such as ALS or FTD. The fact that PHA has been tested in humans and that PLGA is approved by FDA means these NPs will soon be suitable for repurposing.

## 2. Materials and Methods

### 2.1. Materials

Poly (d,l-lactide-co-glycolide) copolymer, PLGA (LACTEL^®^ Absorbable Polymers, inherent viscosity range: 0.95–1.20 dL/g in HFIP, ester terminated (nominal), Madrid, Spain, Part.# B6010-4P) with a 50:50 ratio (PLA:PGA) was used as a biodegradable polymer for the nanoparticle preparation. Polyvinyl alcohol, PVA (Sigma Aldrich, Mw 31.000-50.000, 87–89% hydrolyzed, Cat. Number 363073, Madrid, Spain) was used as a surfactant (stabilizer). Tetrahydrofuran (THF, Scharlab, Barcelona, Spain), methanol (MeOH, VWR) and deonized water (MilliQ system, Milllipore, Madrid, Spain) were used as solvents. PHA-767491 (PHA) was synthesized according to the literature [1] and used as the drug for loaded nanoparticles.

### 2.2. Methods

#### 2.2.1. PLGA Nanoparticles Preparation

Polymeric nanoparticles were prepared by the nanoprecipitation method [20]. For blank or empty NPs, 50 mg of biodegradable polymer (PLGA) was dissolved in 5 mL of THF, with 2 h magnetic stirring, and slowly added dropwise over a 2% *w*/*v* aqueous solution of an emulsion stabilizer, in this case polyvinyl alcohol (PVA). For drug-loaded NPs, the PLGA solution was mixed with 5 or 10 mg of the drug dissolved in 2.5 and 5 mL of THF/MeOH (4:1), respectively, prior to addition to the aqueous solution. For the solvent evaporation, the resulting emulsion was stirred for 2–3 h at room temperature (RT). NPs were collected by centrifugation (Sorvall RC-5C Centrifuge, SS-34 Rotor, Hampton, NY, USA) at 15,000 rpm for 10 min and washed out three times with deionized water. Finally, NPs were frozen at −80 °C in 50 mL Falcon^®^ tubes and lyophilized using a fast-freeze flask (Labconco Corp. 750 mL cat ref #10033692) at −45 °C and 3.2 × 10^−2^ mbar pressure.

#### 2.2.2. Characterization of PLGA Nanoparticles

##### Scanning Electron Microscopy (SEM)

The morphological characterization of the NPs was performed by scanning electron microscopy (SEM) analysis using a JEOL JSM 7600F microscope (Madrid, Spain). Lyophilized samples were fixed onto a sample holder using a carbon adhesive filament and gold-coated for 90 s. SEM images were analyzed for the NP shape.

##### Dynamic Light Scattering (DLS)

The NP size and polydispersity index (PDI) were determined by dynamic light scattering (DLS) using a DynaPro MS/X, Wyatt Inc. instrument (Denbarch, Germany). NPs were re-suspended in deionized water, sonicated for 10 min to improve dispersion homogeneity and diluted to a final working concentration of 5 μg/mL. Every sample was housed in quartz cuvettes and measured 40 times in one run.

##### Confocal Laser Scanning Microscopy (CLSM)

The entrapped drug was imaged using a confocal laser scanning microscope (CLSM) Leica TCS SP5 with a 63x oil immersion objective. Samples were excited at 405 nm laser and the emitted fluorescence was collected at 420–500 nm.

##### Encapsulation Efficiency (*EE%*) and Loading Capacity (*LC%*)

The encapsulation efficiency and drug loading capacity of PHA in the NPs were determined by an indirect method [21] with high-performance liquid chromatography (HPLC) analysis. The column used for the analysis was a SunFire^®^ C18, 3.5 µm, 4.6 × 50 mm^2^ and UV-Vis spectra of the samples were acquired in a Thermo Finnigan Surveyor UV-Vis Plus Detector (Madrid, Spain). Free PHA was quantified by direct injection of the supernatant after the washing steps and the amount of compound entrapped was calculated as the difference between the total amount of PHA (*PHA_total_*) and the amount of free PHA (*PHA_free_*). Encapsulation efficiency (*EE%*) and drug loading capacity (*LC%*) were expressed according to the following Equations (1) and (2):

*EE%* = [(*PHA_total_* − *PHA_free_*)/*PHA_total_*] × 100 (1)

*LC%* = [(*PHA_total_* − *PHA_free_*)/Total NPs] × 100 (2)

#### 2.2.3. Permeability to the CNS

Prediction of the blood-brain barrier (BBB) penetration for the PHA and NPs was evaluated using parallel artificial membrane permeability assay (PAMPA) methodology [22]. Ten commercial drugs purchased from Sigma were used as controls: atenolol, caffeine, enoxacine, hydrocortisone, desipramine, ofloxacine, piroxicam, testosterone, promazine and verapamile. Controls and PHA were dissolved in phosphate buffer saline solution (PBS) at pH 7.4 and ethanol with a 70/30 ratio. NPs were re-suspended in the same solution; 96-well plates from Millipore were used for the assays. The donor plate (Multiscreen^®^ IP Sterile Plate PDVF membrane, pore size 0.45 μM, catalog no. MAIPS4510, Madrid, Spain) was filled with 180 μL of each filtered sample solution after being coated with 4 μL of porcine brain lipid (Avanti Polar Lipids Inc., catalog no. 141101) in dodecane (20 mg/mL). The acceptor plate (Multiscreen^®^ catalog no. MAMCS9610, Madrid, Spain) was filled with 180 μL of the experimental buffer. Then, the donor plate was carefully placed on the acceptor plate to form a sandwich for 2 h and 30 min at 25 °C. During this time, compounds diffuse from the donor plate through the brain lipid membrane into the acceptor plate. After incubation, the donor plate was removed and the concentration of compounds and commercial drugs in the acceptor plate was determined by UV using a 96-well plate reader (Thermoscientific Multiskan spectrum). Every sample was analyzed at three to five wavelengths, in three wells and in two independent runs. For the PHA, results are given as the mean value ± standard deviation (SD) of the two runs. For NPs, results are shown as the relative experimental *Pe* value vs. the free compound.

#### 2.2.4. Neuronal Cell Culture

Human neuroblastoma SH-SY5Y cell line was cultured in Dulbecco’s Modified Eagle Medium (DMEM, Gibco) supplemented with 10% fetal bovine serum (FBS, Gibco) and 1% penicillin/streptomycin (Gibco) at 37 °C and 5% CO_2_ in an incubator.

Cell viability of SH-SY5Y exposed to increasing concentrations of PHA-loaded nanoparticles for 24 h was determined by the 3-(4,5-dimethylthiazol-2-yl)-2,5-diphenyl tetrazolium bromide (MTT) assay. Briefly, 7.5 × 10^4^ cells were seeded onto 96-well plates and treated with different concentrations of PHA-loaded nanoparticles (0.05 µM, 0.1 µM, 0.5 µM and 1 µM of encapsulated PHA). Then, 24 h after the treatment, thiazolyl blue was added to the culture medium at a final concentration of 0.5 mg/mL for at least 4 h at 37 °C. Culture media were removed and 200 µL of DMSO was added to each well to dissolve the formazan crystals. Using a microplate reader (Varioskan Flash Microplate reader, Thermo Scientific, Waltham, MA, USA), absorbance was measured at 595 nm.

For immunofluorescence experiments, SH-SY5Y cells (1.5 × 10^5^ cells/well) were grown in glass coverslips in 24-well plates. Cells were first pre-treated for 2 h with PHA and PHA-loaded NPs at 0.5 µM and after that time ethacrynic acid (EA) was added at a concentration of 40 µM. After 24 h, cells were fixed for 20 min at RT in 4% paraformaldehyde in PBS, washed twice with PBS and stored at 4 °C.

#### 2.2.5. Immunofluorescence Analysis

Cells were permeabilized with 0.25% Triton X-100 (Sigma Aldrich, Madrid, Spain) for 10 min at RT, rinsed with PBS and blocked with 2% BSA (Sigma Aldrich, Madrid, Spain) and 0.1% casein (Sigma Aldrich, Madrid, Spain) for 30 min at RT. Cells were incubated for 1 h at 37 °C with TDP-43 monoclonal antibody (1:800, Proteintech) or phospho TDP antibody (1:500, Proteintech) in 6% BSA, rinsed with PBS and incubated with Alexa Fluor 488 anti-rabbit antibody or Alexa Fluor 561 anti-rabbit antibody (1:600, Jackson Immuno Research). Cell nuclei were stained for 30 min with HCS NuclearMask Deep Red (1:250, Thermo Fisher). Finally, preparations were washed with 1% BSA and 0.1% casein and mounted onto Fluoromount Mounting Medium (Sigma Aldrich). Images were acquired for ∼60 cells per group in *n* = 3 independent experiments using a confocal laser scanning microscope (CLMS) Leica TCS SP5 with 63x oil immersion objective. Quantification and colocalization analyses were performed using ImageJ software (Bethseda, MD, USA, 2020). Mander’s colocalization coefficient M1 was calculated using Coloc 2.

## 3. Results and Discussion

### 3.1. Preparation of PLGA Nanoparticles

PLGA selection as a starting material for NPs was done based on its approval by FDA and European Medicines Agency (EMA) in several delivery systems, leading PLGA-based NPs to be in a good position for further translation of results to clinical settings. PLGA-NPs are non-toxic and display excellent biocompatibility and biodegradability properties [23]. Several methods have been described for the preparation of polymeric NPs depending on their future applications and the chemical properties of the drug to be entrapped [24]. The most common used methods for PLGA-NPs are emulsification solvent evaporation, emulsification solvent diffusion and nanoprecipitation [25]. Emulsification methods such as the double-emulsion technique are frequently used to incorporate hydrophilic drugs, while nanoprecipitation is the most efficient method for the encapsulation of hydrophobic molecules [26]. This technique has been successfully used for PLGA-NPs as nanocarriers for the encapsulation of anti-cancer drugs such as nitrocamphotecin [27] and also for the CNS drug haloperidol [28].

In this work, we first tried the double-emulsion method for PHA encapsulation as it was isolated as a PHA hydrochloride salt, and thus water solubility was high [29]. This first attempt provided very few, large and polydispersed nanoparticles, and required high amounts of polymer and stabilizer which were difficult to remove.

**Figure 2:**
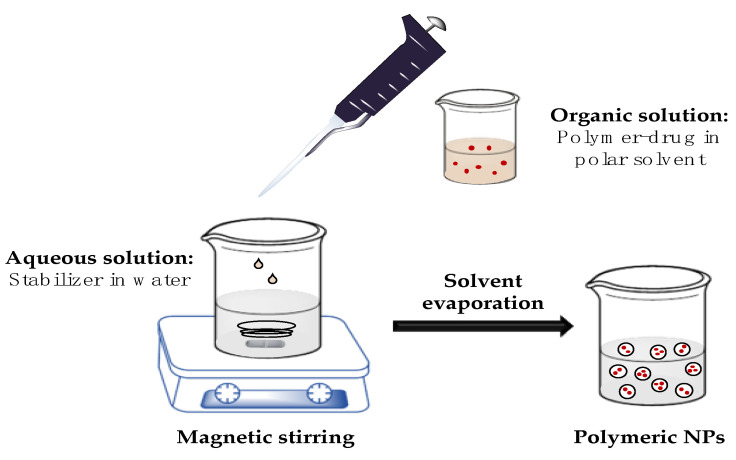
Schematic representation of nanoprecipitation method.

At this point, we decided to use the nanoprecipitation method for PHA-loaded nanoparticles starting from the neutral form of the drug. The nanoprecipitation method, also known as solvent displacement, is a one-step procedure firstly described by Fessi et al. [20]. Briefly, the polymer and the drug are dissolved in a water-miscible organic solution like acetone, methanol or tetrahydrofuran, and this solution is added dropwise and under magnetic stirring to an aqueous phase containing the stabilizer polyvinyl alcohol (PVA) (Figure 2). When the polymer–drug solution is added to the aqueous medium, NPs are formed instantaneously by rapid diffusion of the organic solvent to the aqueous phase and are stabilized thanks to the effect of the surfactant which reduces the interfacial tension between the two phases.

After solvent evaporation and washing steps, nanoparticles were collected and lyophilized to obtain a white powder that could be quantified. Three different formulations were prepared as summarized in Table 1, starting from different quantities of PHA. In all the cases the same amount of PLGA and PVA were used. Control formulations were NPs without drug and in the following two different quantities of PHA (5 and 10 mg) were used. Each formulation was independently repeated two or three times.

As shown, NPs were obtained with great reproducibility in all cases. Furthermore, a greater number of NPs was obtained in presence of the drug (NP-4–8) compared to the blank ones (NP-1–3) due to the drug loading. For further characterization, we selected one formulation of each different condition prepared (0, 5 or 10 mg of PHA). In this case, NP formulations NP-2, NP-4 and NP-7 were used in subsequent analyses.

### 3.2. Characterization of PLGA Nanoparticles

#### 3.2.1. Morphology and Size of Nanoparticles

**Figure 3:**
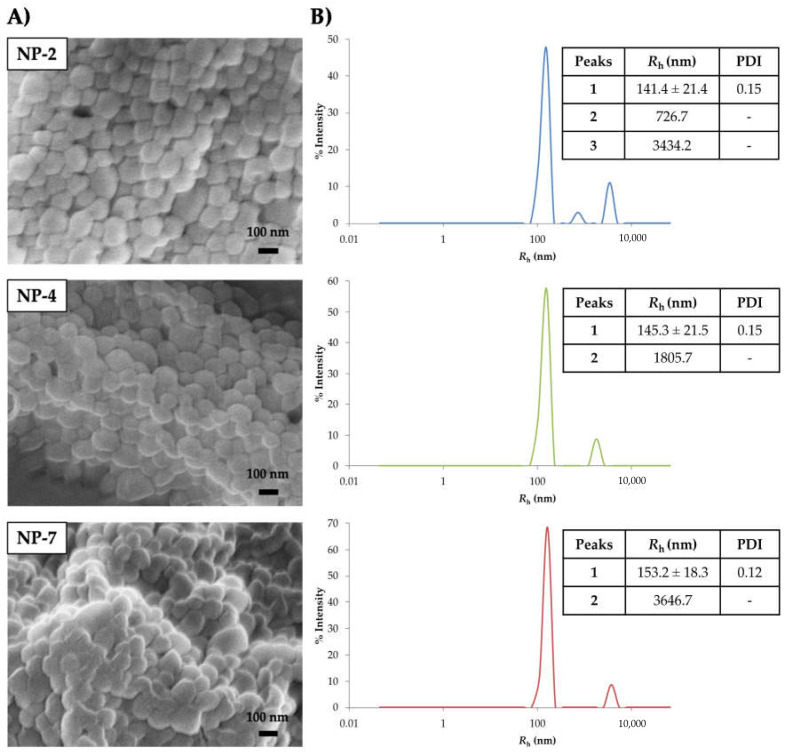
(**A**) Representative SEM (scanning electron microscopy) images of blank nanoparticles (NP-2) and PHA-loaded nanoparticles (NP-4, NP-7). (**B**) Representative size distribution of blank nanoparticles (NP-2) and PHA-loaded nanoparticles (NP-4, NP-7). Results are shown as the mean of 40 measures ± standard deviation (SD). PDI refers to polydispersity of NPs.

A scanning electron microscopy (SEM) technique was used to determine the morphology of the nanoparticles, as it allows us to obtain tridimensional images of particle shape through a direct visualization of the sample [30]. As polymeric NPs are invisible to this technique due to their organic nature, they need to be coated with a thin layer of metal. In this case, NPs were gold-coated to create a conductive layer on the samples. Asa result, SEM images (Figure 3A) of PLGA nanoparticles NP-2, NP-4 and NP-7 indicated a mostly spherical smooth shape for all formulations. This technique also offered information about the size of the NPs, which ranged from 100 to 300 nm.

In order to determine the size of the NPs more accurately, as well as to obtain some information about the degree of dispersity, dynamic light scattering (DLS) was employed [31]. Due to the Brownian motion of particles in solution, light intensity fluctuates in time and these temporary fluctuations depend on particle size, being more frequent for small particles. Finally, the hydrodynamic radius can be obtained using the Stokes–Einstein equation as follows:

*D* = *k*_B_*T*/6π*ηR*_h_ (3)

where, in Equation (3) *D* is the diffusion coefficient measured by DLS, *k*_B_ is the Boltzmann’s constant, *T* is the temperature, *η* is the viscosity of the solvent and *R*_h_ the hydrodynamic radius.

DLS results (Figure 3B) showed a hydrodynamic radius (*R*_h_) for blank and PHA-loaded NPs between 141 and 155 nm. Furthermore, polydispersity index (PDI) was calculated. As the polydispersity index is a measure of the homogeneity of a particle solution, the PDI values obtained, which were less than 0.15, may show a monodispersed particle suspension with a single size of NPs.

These results (particle shape, size and PDI values) are comparable to those of nanoparticles studied for drug delivery to the brain, such as superoxide dismutase 1 (SOD1) PLGA-NPs (*D*_h_ = 291 nm, PDI = 0.12) which were studied for the treatment of cerebral ischemia [18,19,20,21,22,23,24,25,26,27,28,29,30,31,32], supporting their great potential for the treatment of neurological conditions.

#### 3.2.2. Efficiency of Drug Encapsulation

**Figure 4:**
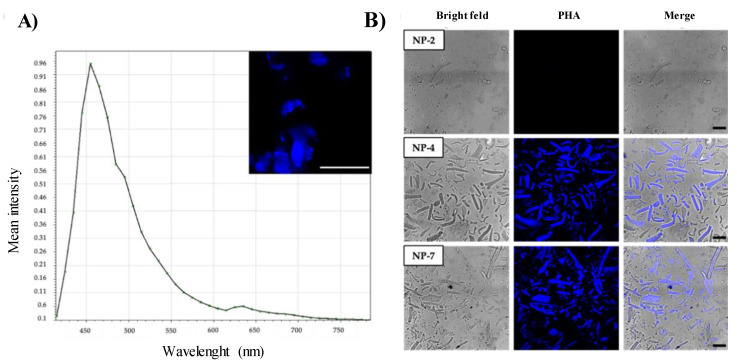
(**A**) Emission spectrum of PHA. (**B**) Representative confocal images of blank nanoparticles (NP-2) and PHA-loaded NPs (NP-4 and NP-7). Scale bars: 20 μm.

Based on the intrinsic fluorescence of PHA, NPs were imaged using confocal laser scanning microscopy (CLSM) to determine if the compound is entrapped [33]. This imaging technique can be used to collect and detect the emitted light from fluorescent particles and to obtain 3D images of the sample from different optical planes with high resolution. First, the emission spectrum of PHA was recorded by scanning a wavelength range of 415–795 nm using the 405 nm laser. We observed that PHA had a maximum emission peak at 455 nm (Figure 4A). Then, NPs were suspended in deionized water and fluorescence was collected between 420–500 nm. PHA-loaded NPs showed fluorescence (NP-4 and NP-7) while the blank ones (NP-2) did not (Figure 4B), demonstrating that PHA was successfully encapsulated into the nanoparticles. Furthermore, all PHA-loaded NPs showed similar fluorescence intensity which indicates that an equivalent amount of PHA was encapsulated.

Entrapment efficacy is an extremely important parameter to consider when preparing nanoparticles as drug delivery systems. This can be measured by two parameters: entrapment efficiency (*EE%*) and loading capacity (*LC%*) [34]. The entrapment efficiency is a measure of the drug that has been successfully encapsulated in the NPs, while the drug loading capacity reflects the mass ratio of drug to NPs. Determination of the precise drug content is not easy because NPs are colloidal systems and are different from one drug to another. Thus, encapsulation efficiency of drugs varies from 6% to 90% for dexamethasone and paclitaxel, respectively [35,36]. Among the different methods that can be used to determine these two parameters [21], in this case we used an indirect method based on HPLC determinations (described in Materials and Methods) and following Equations (1) and (2). In the first step, different solutions of free PHA were used to correlate the peak area with the drug concentration and a good correlation between these values was obtained: Area = 2 × 10^7^ [PHA] + 93.5952 (R^2^ = 0.9885) (Figure S1). Then, the supernatant after the washing steps for each formulation was analyzed and, following this equation, free PHA was quantified. The amount of entrapped PHA was calculated as the difference between the initial amount of PHA used for NPs formulations and the amount of PHA in supernatant. Results are collected in Table 2.

In all cases, the *EE%* ranged from 12% to 18%, with a LC% between 2% and 4%. These values are in agreement or slightly better than in other PLGA-based NPs where the *LC%* is around 1% [19].

### 3.3. Permeability to the CNS

**Figure 5:**
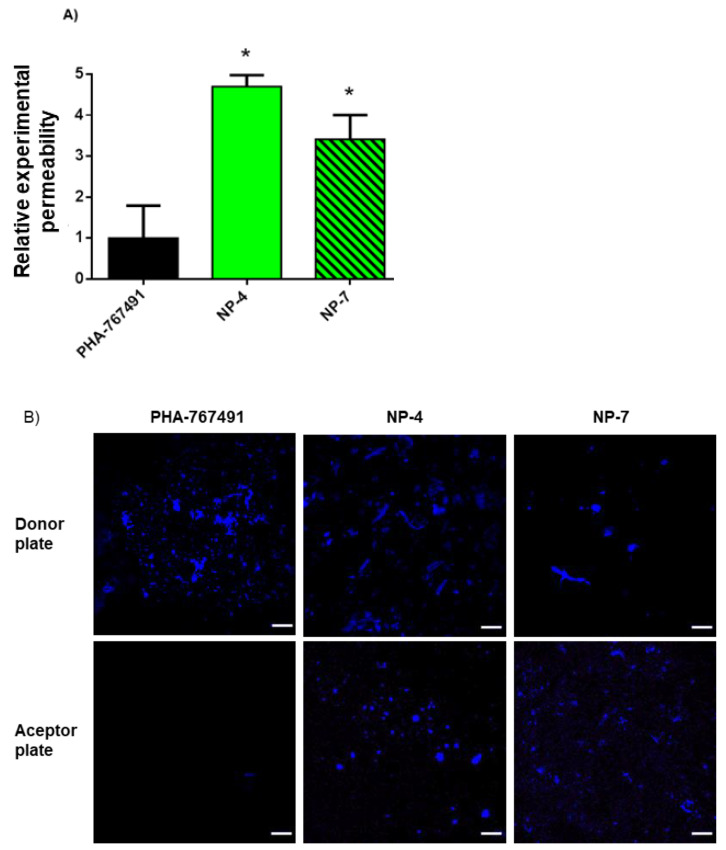
Predicted permeability through the BBB (blood–brain barrier) assayed by PAMPA (parallel artificial membrane permeability assay) methodology. (**A**) Relative experimental permeability of PHA-loaded NPs vs. free PHA. Results are shown as the mean ± SD of two independent experiments. (* *p* < 0.05 significantly different from free PHA). (**B**) CLSM (confocal laser scanning microscopy) images of donor and acceptor plates after incubation. Scale bars: 20 µm.

Brain penetration is necessary in order to reach the desired target and exert its therapeutic effect. In this work, parallel artificial membrane permeability assay (PAMPA) methodology was performed using porcine lipid to emulate the human BBB to determine the permeability of free PHA and PHA-loaded nanoparticles by passive diffusion [37,38]. Ten commercial drugs with known permeability into the CNS were used as controls and a good correlation between the experimental and reported values was obtained: *Pe* (exp.) = 1.27 (bibl.) + 0.5332 (R^2^ = 0.9852) (Table S1 and Figure S2). From this equation and following the pattern described in the literature for human BBB permeability [39], *Pe* values were calculated considering CNS+ when they presented a permeability > 5.61 × 10^−6^ cm s^−1^. Based on these results, PHA was not able to cross the BBB as was previously described. In contrast, PHA permeability increased 3–4-fold when it was loaded in the PLGA-NPs (Figure 5A).

To support these results and taking advantage of the intrinsic fluorescence of PHA, donor and acceptor plates from PAMPA assays were analyzed by confocal laser scanning microscopy. We then observed a higher amount of PHA in the acceptor plates for PHA-loaded NPs compared to free (non-encapsulated) PHA (Figure 5B). Interestingly, only single spherical nanoparticles were found in the acceptor plates, meanwhile heterogeneous nanoparticles in shape and size remained in the donor plates. These results confirm that free PHA is not able to cross the blood-brain barrier (BBB) but PHA-loaded NPs have an increased permeability. The change in the physico-chemical properties of the PHA surface produced by the nanoencapsulation enhanced the BBB penetration by passive transport.

### 3.4. Neuroprotective Efficacy of PHA-Loaded PLGA Nanoparticles in a Cell Model of TDP-43 Phosphorylation

We recently reported that brain-permeable purine-based CDC7 inhibitors are able to rescue neuronal cells from the death induced by ethacrynic acid (EA) [40]. EA induced TDP-43 phosphorylation in human neuroblastoma cell line SH-SY5Y by glutathione depletion, decreasing cell viability. In these conditions, when the cell cultures were pretreated for 1 h with different kinase inhibitors able to decrease TDP-43 phosphorylation, we showed cell survival preservation [40,41].

**Figure 6:**
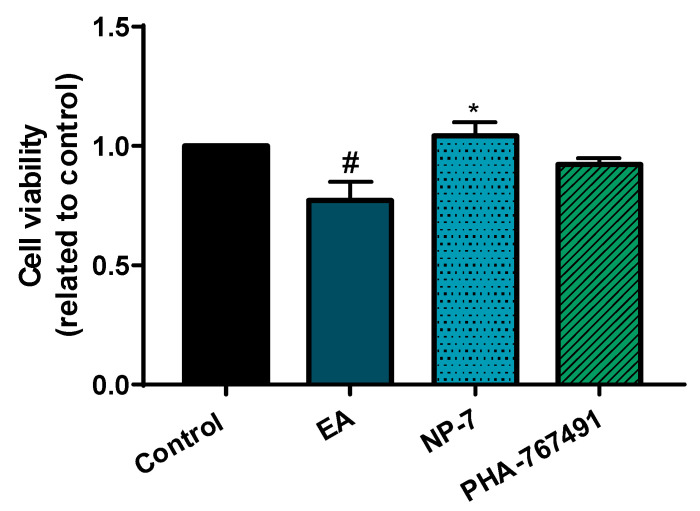
EA (ethacrynic acid)-induced neurodegeneration model. Cell viability after treatment with NP-7 or PHA at 0.5 µM for 2 h and subsequently with EA (40 µM) was measured by the 3-(4,5-dimethylthiazol-2-yl)-2,5-diphenyl tetrazolium bromide (MTT) test. Data represent the mean ± SEM. (* *p* < 0.05, significantly different from SH-SY5Y EA-treated cells, # *p* < 0.05 significantly different from control).

Here, and to show the therapeutic potential of PHA-loaded PLGA nanoparticles, we evaluated their neuroprotective efficacy in the abovementioned model. Free PHA was used for comparison in the same assay and the dose was selected based in its IC_50_ value over CDC7 [1]. NP-7 formulation was used in this study at a concentration of 0.5 µM which was equivalent to the quantity of free PHA used as control. Previously, a study was done to show that the selected NP-7 concentration does not interfere with cell viability (Figure S3). In this experiment, we observed how the NP-7 formulation significantly protects neuronal cells from death induced by EA (Figure 6).

**Figure 7:**
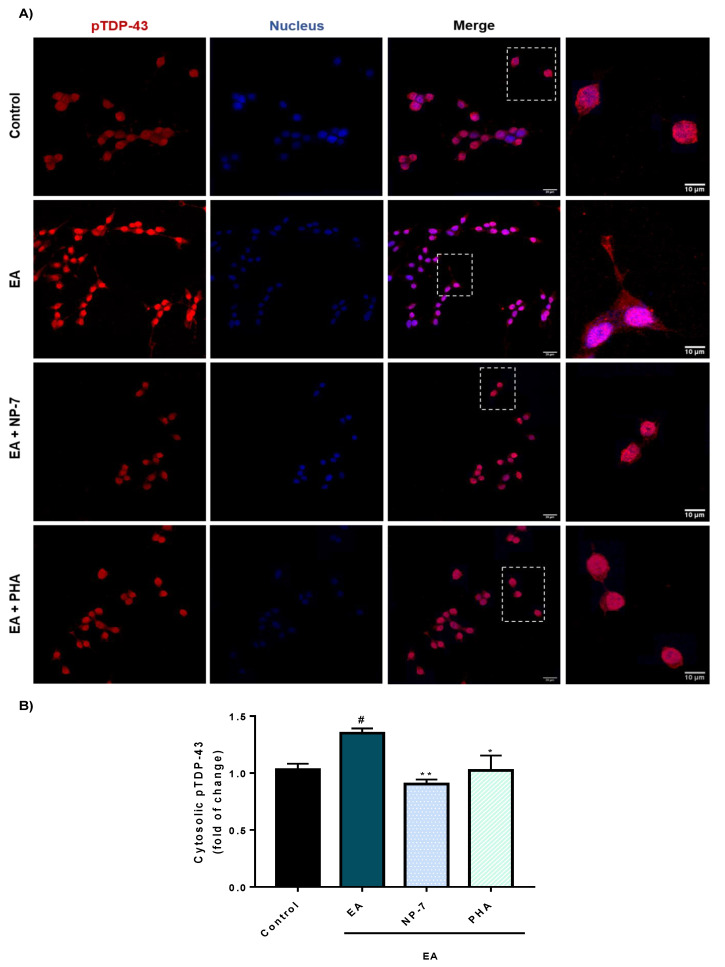
Immunofluorescence analysis of the TDP-43 phosphorylation in SH-SY5Y cells exposed to EA after PHA and NP-7 treatment. (**A**) Cells were pre-treated with PHA (0.5 µM) or NP-7 (0.5 µM) and exposed to EA (40 µM). NP-7 concentration was calculated referred to encapsulated PHA so cells were exposed to the same amount of PHA. Cytosolic pTDP-43 levels were assessed by confocal laser scanning microscopy. Scale bars: 10 µM and 20 µM. (**B**) Quantification of fluorescence intensity of cytosolic pTDP-43 was determined in at least 50 different cells from 3 separate wells (*n* = 3). Data represent the mean ± SEM (magnification 63×). (* *p* < 0.05, ** *p* < 0.005 significantly different from SH-SY5Y EA-treated cells, # *p* < 0.05, significantly different from control).

**Figure 8:**
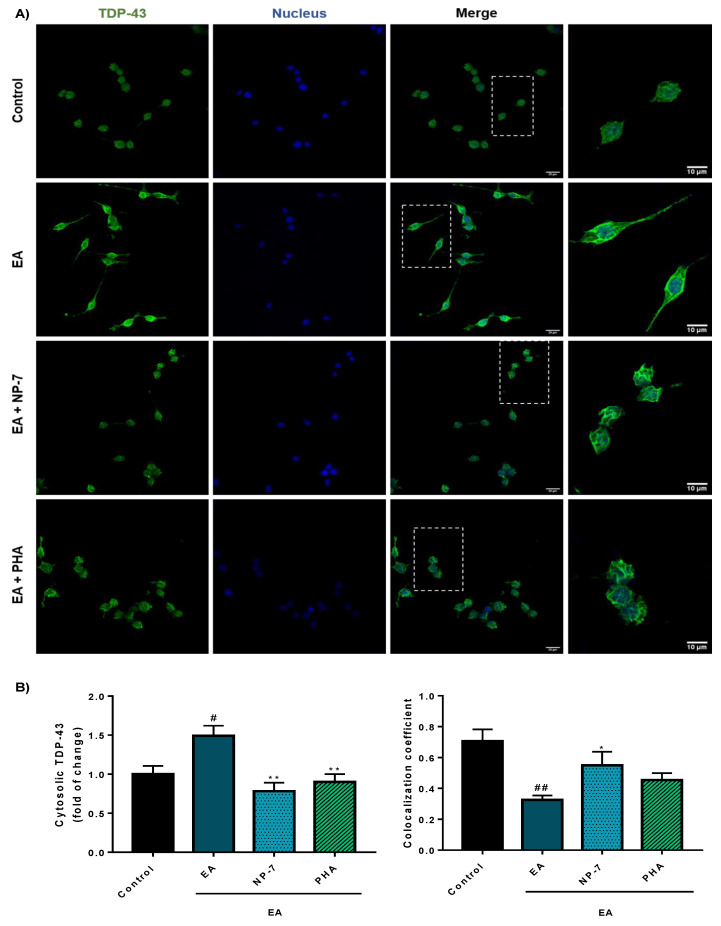
Immunofluorescence analysis of the subcellular localization of TDP-43 in SH-SY5Y cells exposed to EA after PHA and NP-7 treatment. (**A)** Cells were pre-treated with PHA (0.5 µM) or NP-7 (0.5 µM) and exposed to EA (40 µM). NP-7 concentration was calculated, referring to encapsulated PHA, so cells were exposed to the same amount of PHA. TDP-43 localization was assessed by confocal laser scanning microscopy. Scale bar: 10 µM and 20 µM. (**B**) Quantification of cytosolic TDP-43 (right) and colocalization analysis by calculating Mander’s colocalization coefficient which indicates the percentage of total TDP-43 inside the nucleus (left). At least 50 different cells from 3 separate wells (*n* = 3) were analyzed. Data represent the mean ± SEM (magnification 63×). (* *p* < 0.05, ** *p* < 0.005 significantly different from SH-SY5Y EA-treated cells, # *p* < 0.05, ## *p* < 0.005 significantly different from control).

Furthermore, TDP-43 pathology is better modulated by PHA-loaded NPs than with PHA alone. Thus, phosphorylation of TDP-43 decreased (Figure 7) together with aberrant cytoplasmic localization (Figure 8).

## 4. Conclusions

Biodegradable polymeric nanoparticles based on PLGA were obtained by the nanoprecipitation method and were loaded with PHA, a potent CDC7 inhibitor with great potential for some neurodegenerative diseases but with low brain permeability. The methodology here reported shows a way to overcome the lack of CNS penetration of PHA, offering an interesting approach for further development as a potential therapy for TDP-43 proteinopathies and other neurological disorders. Furthermore, these new PHA-loaded NPs have improved the reduction of pTDP-43 levels in a cellular model. PLGA-NPs have shown once more to be a useful technology to overcome the BBB for CNS-active small molecules.

## Figures and Tables

**Table 1:** Formulations for blank and drug-loaded nanoparticles preparation.

Formulations	Raw Materials			Final NPs (mg)
	PHA (mg)	PLGA (mg)	PVA (mg)	
NP-1	-	51.4	400.0	31.0
NP-2	-	52.4	400.5	32.6
NP-3	-	52.5	401.5	24.4
NP-4	5.0	50.2	400.1	32.8
NP-5	5.6	51.7	403.6	42.4
NP-6	10.4	51.5	401.6	51.8
NP-7	10.2	50.5	402.4	41.1
NP-8	10.1	51.0	401.5	46.8

**Table 2:** Encapsulation efficiency and loading capacity of PLGA (poly (lactic-co-glycolic acid)) nanoparticles.

Formulations	PHA_total_ (mg)	Total NPs (mg)	PHA_entrapped_ (mg)	*EE%*	*LC%*
NP-4	5.0	32.8	0.7	14	2
NP-5	5.6	42.4	0.8	14	2
NP-6	10.4	51.8	1.6	15	3
NP-7	10.2	41.1	1.2	12	3
NP-8	10.1	46.8	1.9	18	4

## Data Availability

The data presented in this study are available online Supplementary Materials.

## Supplementary Materials

The following are available online at https://www.mdpi.com/1999-4923/13/2/180/s1. Figure S1: HPLC analysis. Linear correlation between the area under the curve and the concentration of free PHA, Figure S2: PAMPA-BBB assay. Linear correlation between experimental and reported permeability of ten commercial drugs used for the experiment validation, Figure S3: Cell viability of SH-SY5Y in the presence of NP-7 at 0.05, 0.1, 0.5 and 1 μM of encapsulated PHA. Cell viability was measured 24 h after treatment by MTT assay. Data represent the mean ± SEM of 3 different experiments. (** *p* < 0.01 significantly different from control), Table S1: PAMPA-BBB assay permeability (reported and experimental) of ten commercial drugs used as controls and experimental permeability of PHA with its predictive penetration into the CNS.
