# Supplementary material for: Increasing Brain Permeability of PHA-767491, a Cell Division Cycle 7 Kinase Inhibitor, with Biodegradable Polymeric Nanoparticles
Source: Pharmaceutics. 2021 Jan 28;13(2):180. doi: 10.3390/pharmaceutics13020180 (PMC7912371; doi:10.3390/pharmaceutics13020180)
Supplement: Supplementary file 1 [file pharmaceutics-13-00180-s001.pdf]

pharmaceutics

MDPI

Supplementary Materials: Increasing Brain Permeability of PHA-767491, a Cell Division Cycle 7 kinase Inhibitor, with Biodegradable Polymeric Nanoparticles

Elisa Rojas-Prats, Carlota Tosat-Bitrián, Loreto Martínez-González, Vanesa Nozal, Daniel I. Pérez and Ana Martínez

Table 1. PAMPA-BBB assay permeability (reported and experimental) of ten commercial drugs used as controls and experimental permeability of PHA with its predictive penetration into the CNS. a) Reference [1]. b) Data represent the mean ◎ SD of 2 independent experiments. c) Experimental prediction of BBB permeation by passive diffusion: CNS+: high BBB permeability, CNS-: low BBB permeability, CNS+/ CNS -: intermediate BBB permeability.

Compound	Bibl. [a]	*Pe* (10^{-6} cm s^{-1}) [b]	Prediction [c]
Atenolol	0.8	0.3 ± 0.2	CNS -
Caffeine	1.3	0.7 ± 0.0	CNS -
Desipramine	12.0	11.1 ± 0.8	CNS +
Enoxacin	0.9	0.3 ± 0.2	CNS -
Hydrocortisone	1.9	2.0 ± 0.3	CNS +/ CNS -
Ofloxacin	0.8	0.2 ± 0.1	CNS -
Piroxicam	2.5	0.8 ± 0.1	CNS +/ CNS -
Promazine	8.8	10.0 ± 1.4	CNS +
Testosterone	17.0	14.4 ± 2.6	CNS +
Verapamil	16.0	13.2 ± 2.4	CNS +
PHA		1.9 ± 0.1	CNS -

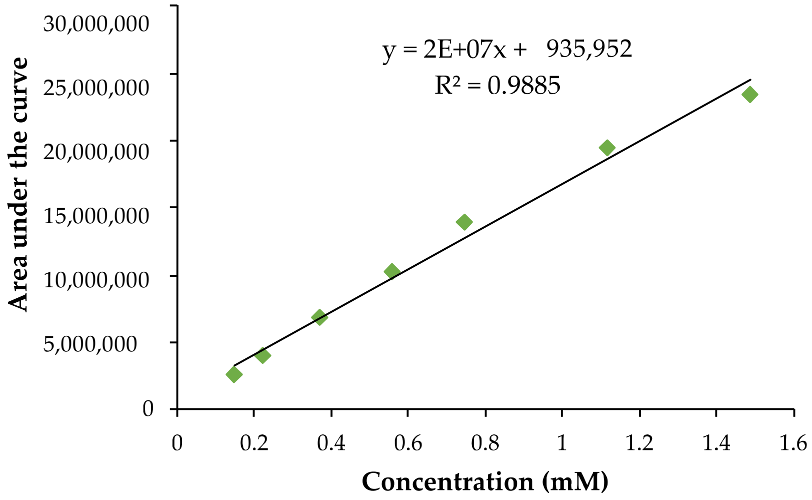

Figure S1. HPLC analysis. Linear correlation between the area under the curve and the concentration of free PHA.

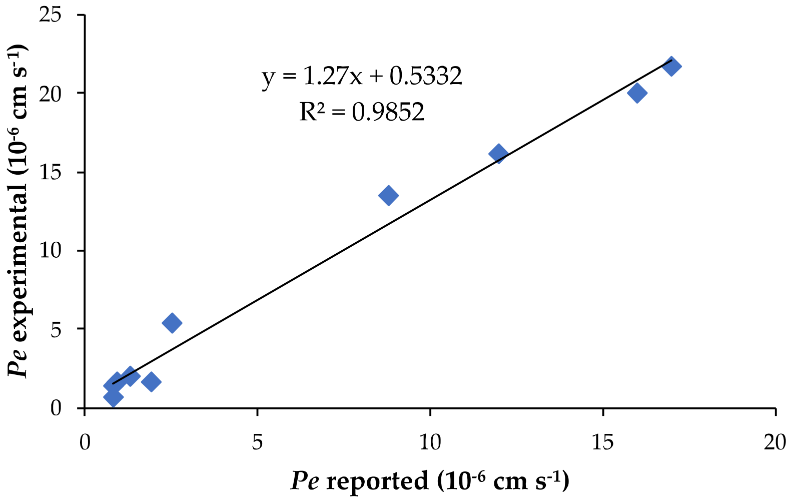

Figure S2. PAMPA-BBB assay. Linear correlation between experimental and reported permeability of ten commercial drugs used for the experiment validation.

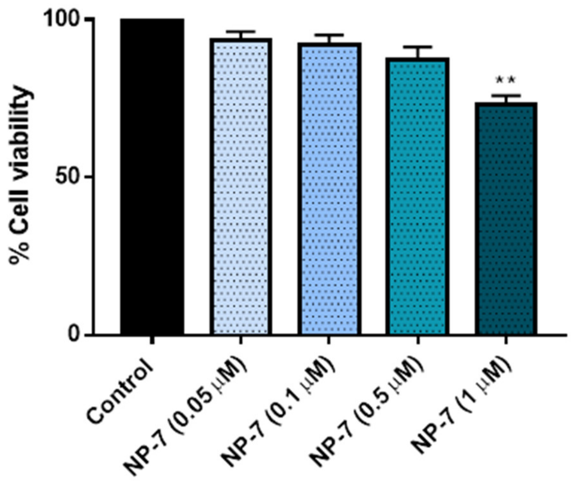

Figure S3. Cell viability of SH-SY5Y in the presence of NP-7 at 0.05, 0.1, 0.5 and 1 µM of encapsulated PHA. Cell viability was measured 24 h after treatment by MTT assay. Data represent the mean ± SEM of 3 different experiments. (**$p < 0.01$ significantly different from control).

References

1. Di, L.; Kerns, E. H.; Fan, K.; McConnell, O. J.; Carter, G. T., High throughput artificial membrane permeability assay for blood-brain barrier. *Eur. J. Med. Chem.* **2003,** *38* (3), 223–232.
